# Supplementary figures and images for: Mapping Atlantic rainforest degradation and regeneration history with indicator species using convolutional network
Source: PLoS One. 2020 Feb 28;15(2):e0229448. doi: 10.1371/journal.pone.0229448 (PMC7048271; doi:10.1371/journal.pone.0229448)

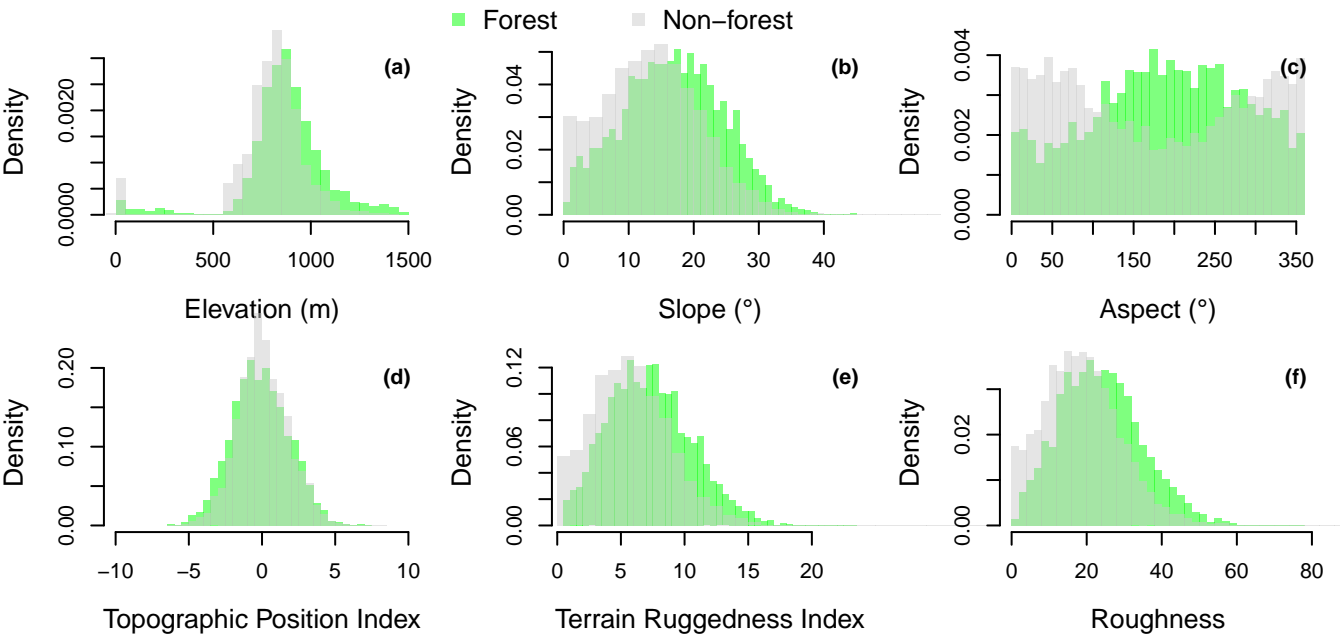

Supplement: S1 Fig — The five elevation related indices consider the eight neighbor pixels and are slope (°); aspect (compass direction facing slope in °, where 0 is the North); topographic position index (elevation of the pixel in relation to the mean elevation of the neighbour pixels); terrain ruggedness index (sum of the change in elevation between the pixels and its 8 neighbors cells); and, finally roughness (difference between the maximum and the minimum value of a cell and its 8 surrounding cells) [31, 32]. (PDF) [file pone.0229448.s001.pdf]

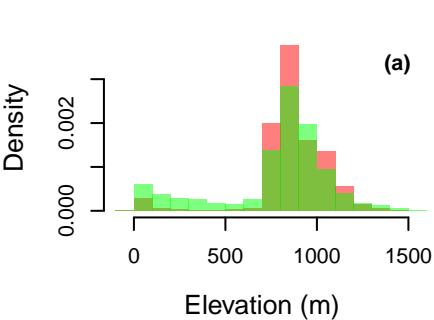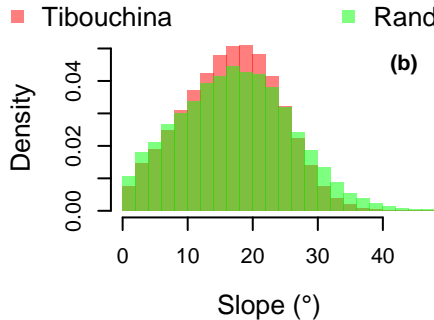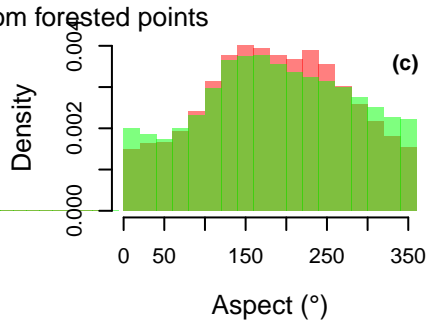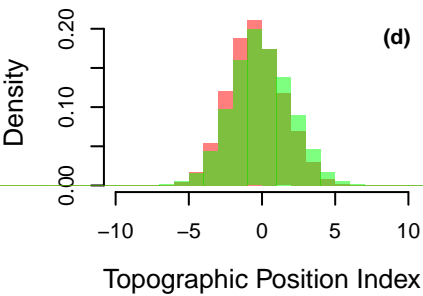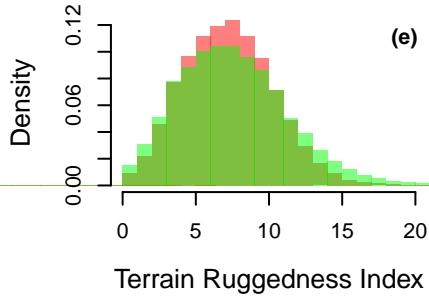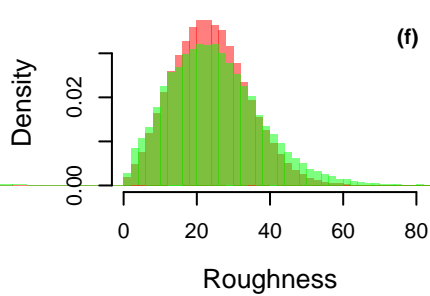

Supplement: S2 Fig — Elevation-related variables were extracted for T. pulchra dominance polygon centroids (n = 461355) and from randomly distributed natural forests points without T. pulchra dominance (n = 50000). (PDF) [file pone.0229448.s002.pdf]

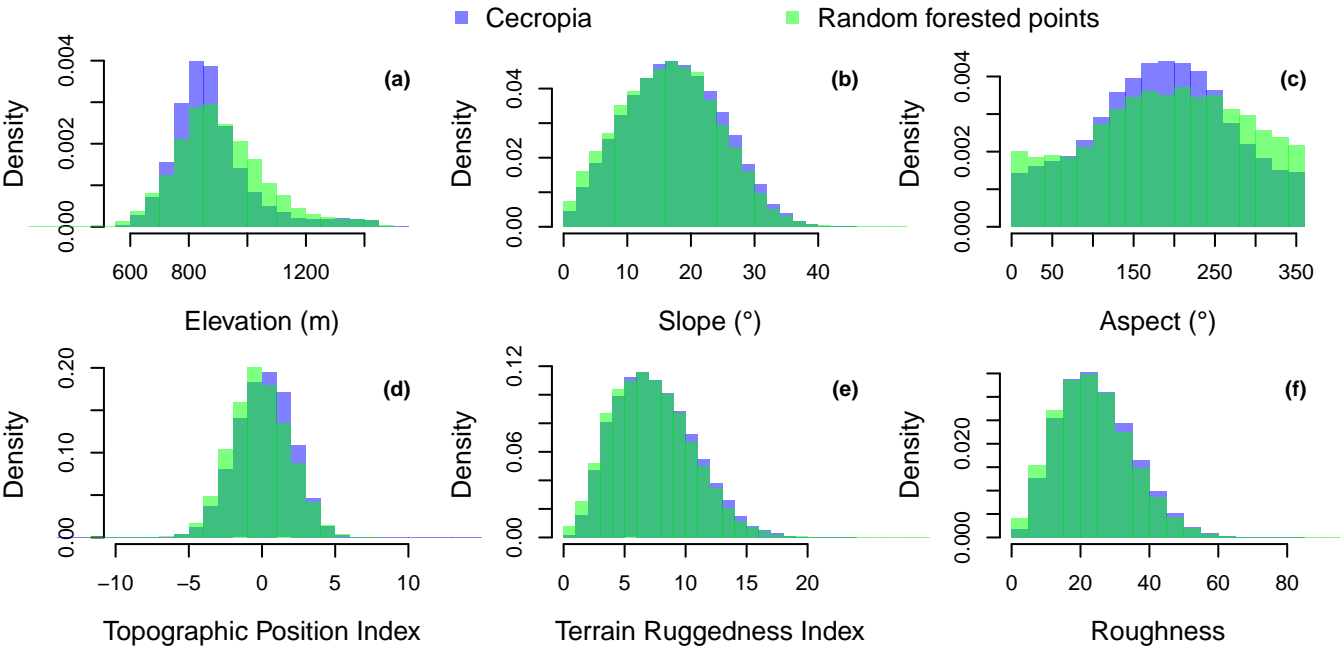

Supplement: S3 Fig — Elevation-related variables were extracted for C. hololeuca dominance polygon centroids (n = 169542) and from randomly distributed natural forests points without C. hololeuca dominance (n = 11717). (PDF) [file pone.0229448.s003.pdf]

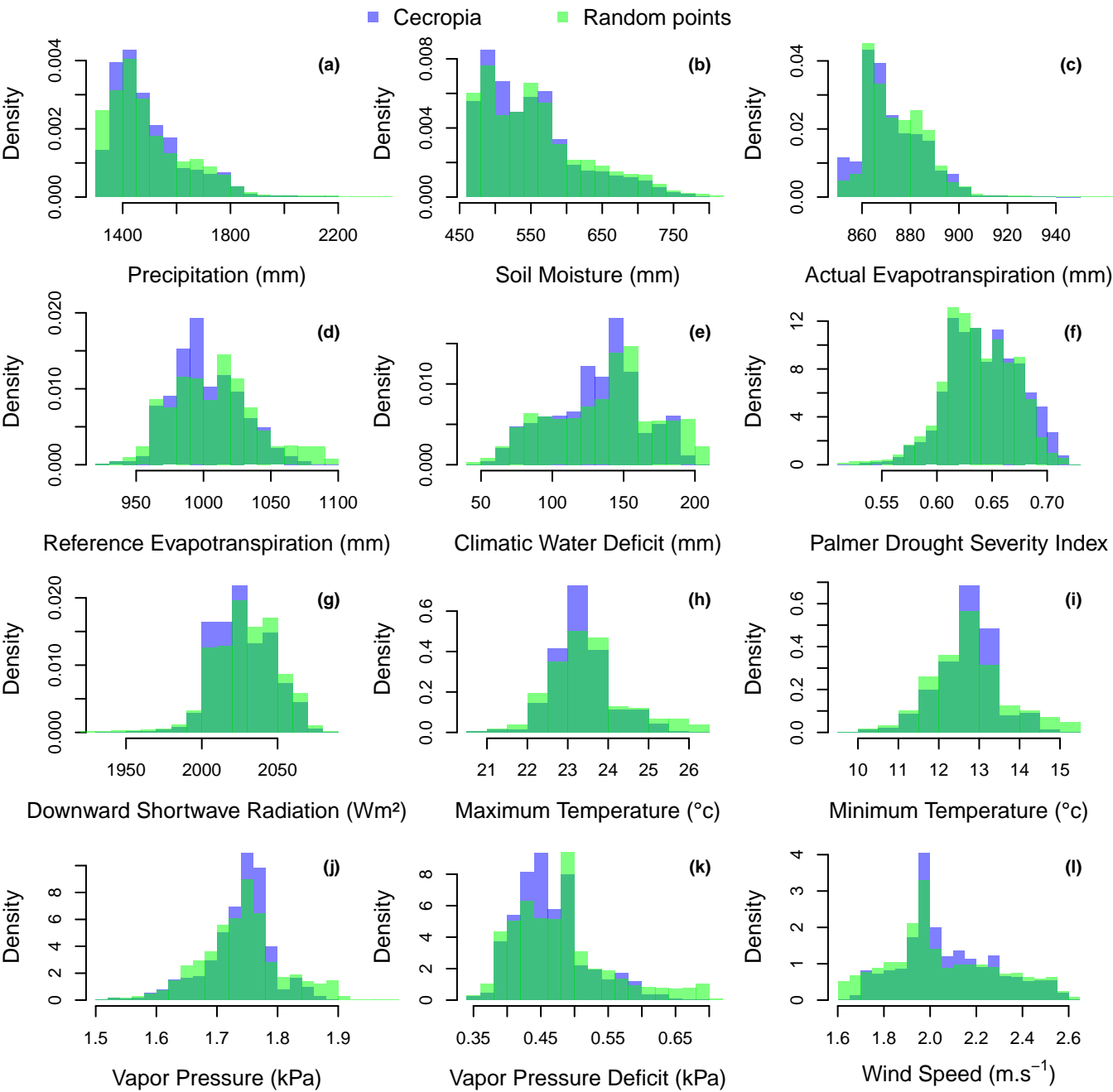

Supplement: S4 Fig — Climate variables were extracted for C. hololeuca dominance polygon centroids (n = 169542) and from randomly distributed natural forests points without C. hololeuca dominance (n = 11717). (PDF) [file pone.0229448.s004.pdf]
